# Role of Nutrition in the Management of Hepatic Encephalopathy in End-Stage Liver Failure

**DOI:** 10.1155/2010/489823

**Published:** 2010-12-22

**Authors:** Chantal Bémeur, Paul Desjardins, Roger F. Butterworth

**Affiliations:** ^1^Neuroscience Research Unit, CHUM, Saint-Luc Hospital, University of Montreal, 1058 St-Denis Street, Montreal, QC, Canada; ^2^Department of Nutrition, University of Montreal, Montreal, QC, Canada

## Abstract

Malnutrition is common in patients with end-stage liver failure and hepatic encephalopathy, and is considered a significant prognostic factor affecting quality of life, outcome, and survival. The liver plays a crucial role in the regulation of nutrition by trafficking the metabolism of nutrients, their distribution and appropriate use by the body. Nutritional consequences with the potential to cause nervous system dysfunction occur in liver failure, and many factors contribute to malnutrition in hepatic failure. Among them are inadequate dietary intake, malabsorption, increased protein losses, hypermetabolism, insulin resistance, gastrointestinal bleeding, ascites, inflammation/infection, and hyponatremia. Patients at risk of malnutrition are relatively difficult to identify since liver disease may interfere with biomarkers of malnutrition. The supplementation of the diet with amino acids, antioxidants, vitamins as well as probiotics in addition to meeting energy and protein requirements may improve nutritional status, liver function, and hepatic encephalopathy in patients with end-stage liver failure.

## 1. Introduction

Malnutrition is a common complication of end-stage liver failure (cirrhosis) and is an important prognostic indicator of clinical outcome (survival rate, length of hospital stay, posttransplantation morbidity, and quality of life) in patients with cirrhosis. Several studies have evaluated nutritional status in patients with liver cirrhosis of different etiologies and varying degrees of liver insufficiency [1, 2] leading to a consensus of opinion that malnutrition is recognizable in all forms of cirrhosis [3] and that the prevalence of malnutrition in cirrhosis has been estimated to range from 65%–100% [4, 5]. The causes of malnutrition in liver disease are complex and multifactorial.

The present paper reviews the role of nutrition in relation to the management of hepatic encephalopathy (HE), a major neuropsychiatric complication of end-stage liver failure. Nutritional consequences of liver failure with the potential to cause central nervous system dysfunction are reviewed. In particular, the roles of dietary protein (animal versus vegetable), branched-chain amino acids, dietary fibre, probiotics, vitamins and antioxidants, minerals (zinc, magnesium) as well as L-carnitine in relation to HE are discussed. An update of the impact of nutritional supplementation on the management of HE is included.

## 2. Malnutrition in Liver Disease

The functional integrity of the liver is essential for nutrient supply (carbohydrates, fat, and proteins), and the liver plays a fundamental role in intermediary metabolism. For example, the liver regulates the synthesis, storage, and breakdown of glycogen, and hepatocytes express enzymes that enable them to synthesize glucose from various precursors such as amino acids, pyruvate, and lactate (gluconeogenesis). In addition, the liver is a major site of fatty acid breakdown and triglyceride synthesis. The breakdown of fatty acids provides an alternative source of energy when glucose is limited during, for example, fasting or starvation. The liver also plays a crucial role in the synthesis and degradation of protein. Protein synthesis by the liver is influenced by the nutritional state, as well as by hormones and alcohol. 

The liver plays a central role in the regulation of nutrition by trafficking the metabolism of nutrients, and many factors disrupt this metabolic balance in end-stage liver failure. Consequently, when the liver fails, numerous nutritional problems occur (Table 1). Several factors contribute to malnutrition in liver failure including inadequate dietary intake of nutrients, reduction in their synthesis or absorption (diminished protein synthesis, malabsorption), increased protein loss, disturbances in substrate utilization, a hypermetabolic state as well as increased energy-protein expenditure and requirements. Because of decreased glycogen stores and gluconeogenesis [6], energy metabolism may shift from carbohydrate to fat oxidation [7] while insulin resistance may also develop. Consequently, liver cirrhosis frequently results in a catabolic state resulting in a lack of essential nutrients. 

It has been estimated that at least 25% of patients with liver cirrhosis experience HE during the natural history of the disease. HE is more frequent in patients with more severe liver insufficiency and in those with spontaneous or surgically created portal-systemic shunts. Whether or not malnourished patients are more prone to develop HE has not been clearly established, but could be anticipated based on several factors. Firstly, malnutrition tends to be more common in patients with advanced liver disease, and HE is more likely in this group. Secondly, nutritional deficits such as decreased lean body mass (muscle is important in ammonia uptake) and hypoalbuminemia (which increases free tryptophan levels) could promote HE [8].

## 3. Factors Contributing to Malnutrition in Cirrhosis

**Figure 1:**
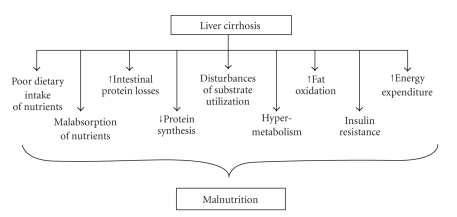
Factors contributing to malnutrition in end-stage liver failure.

A range of factors are known to contribute to malnutrition in cirrhosis. These factors include (Figure 1) the following.

### 3.1. Inadequate Dietary Intake

Cirrhotic patients may unintentionally consume a low energy diet, an observation that is attributed to several factors including loss of appetite [9], anorexia, nausea, vomiting, early satiety, taste abnormalities, poor palatability of diets, reflux disease [10, 11], and impaired expansion capacity of the stomach [9].

### 3.2. Inadequate Synthesis or Absorption of Nutrients

The cirrhotic liver may inadequately synthesize proteins and has diminished storage capacity and an impaired enterohepatic cycle. In addition, portal hypertensive enteropathy may lead to impaired absorption of essential nutrients. Moreover, pancreatic insufficiency, cholestasis, and drug-related diarrhea may all contribute to malabsorption in liver disease.

### 3.3. Increased Protein Losses

Loss of proteins and minerals may result from complications of cirrhosis or from iatrogenic interventions such as the use of diuretics for the treatment of ascites and fluid retention as well as from the use of lactulose for the management of HE. Other potentially important causes of increased protein losses are blood loss from oesophageal and gastric varices and from the intestinal lumen due to ulcers or portal enteropathy.

### 3.4. Hypermetabolic State/Increased Energy-Protein Expenditure and Requirements

The hyperdynamic circulation in cirrhosis leads to systemic vasodilation and an expanded intravascular blood volume. As a direct effect, a higher cardiac blood volume and therefore a greater use of macro- and micronutrients is a common cause of high energy expenditure and demand. Furthermore, the inability of the damaged liver to adequately clear activated proinflammatory mediators such as cytokines may promote the development of an inflammatory response with an increase in both energy expenditure and protein catabolism [12]. It has been suggested that elevated pro- and anti-inflammatory cytokine levels have the potential to result in hypermetabolism in cirrhosis [13, 14].

### 3.5. Insulin Resistance

Insulin resistance and diabetes mellitus are common in patients with liver cirrhosis [15, 16]. Hyperinsulinemia and hyperglucagonemia are frequently present in cirrhotic patients where glucagon is disproportionately increased resulting in an elevated glucagon/insulin ratio. There is also impairment of glucose homeostasis due to hepatic insulin resistance characterized by altered gluconeogenesis, low glycogen stores, and impaired glycogenolysis [15, 16].

### 3.6. Gastrointestinal Bleeding

Bleeding esophageal varices as a consequence of portal hypertension are frequent and severe complications of liver cirrhosis. Gastrointestinal bleeding is also a precipitating factor in HE and may accelerate progression of malnutrition in cirrhotic patients.

### 3.7. Ascites

Impaired expansion capacity of the stomach due to the presence of clinically evident ascites may lead to an inadequate intake of nutrients [9], and cirrhotic patients with ascites often report early satiety and subsequent decreased oral intake which may result in significant weight loss [17].

### 3.8. Inflammation/Infection

Malnourished patients with cirrhosis are prone to the development of inflammation and sepsis and their survival may be further shortened by these complications. There is a significant negative correlation between plasma levels of proinflammatory cytokines such as tumor necrosis factor-alpha (TNF-*α*) and nutrient intake [18]. In order to reduce intestinal bacterial translocation and to improve gut immune function, it has been proposed that pre- and probiotics be added to the diet [19].

### 3.9. Hyponatremia

Hyponatremia is a common complication of patients with advanced liver disease [20] and is an important predictor of short-term mortality. Hyponatremia is also an important pathogenic factor in patients with HE. Cirrhotic patients have abnormal sodium and water handling that may lead to refractory ascites. These patients retain sodium, and dilutional hyponatremia may develop, characterized by reduced serum sodium. In such situations, saline infusion should be avoided and it has been suggested that sodium intake should not exceed 2 g [21].

## 4. Assessment of Nutritional Status in End-Stage Liver Failure

The nutritional assessment of the cirrhotic patient begins with the dietary history that should focus on nutritional intake and assessment of recent weight loss. However, altered mental status may preclude obtaining a meaningful history, and interviewing family members may be helpful.

Liver disease may interfere with biomarkers of malnutrition such as albumin, making it difficult to identify subjects at risk of malnutrition and to evaluate the need for nutritional intervention. Furthermore, anthropometric and bioelectrical impedance analysis may be biased by the presence of edema or ascites associated with liver failure. Body mass index (BMI), an index of nutritional status, may also be overvalued in patients with edema and ascites. Careful interpretation of nutritional data using these techniques in the presence of these complications is therefore required.

Generally accepted methods for assessing the clinical status and severity of disease in cirrhotic patients are the Child-Pugh-Turcotte classification [22] and the model for end-stage liver disease (MELD) [23, 24]. Unfortunately, these systems do not include an assessment of nutritional status in spite of the fact that malnutrition plays an important role in morbidity and mortality in end-stage liver failure. The omission of nutritional assessment results no doubt from the heterogeneous nature of the nutritional deficits in this population. 

Subjective Global Assessment (SGA) and anthropometric parameters are the methods that are frequently used to evaluate nutritional status in end-stage liver failure [25]. SGA collects clinical information through history-taking, physical examination, and recent weight change and is considered to be reliable since it is minimally affected by fluid retention or the presence of ascites. The use of anthropometric parameters which are not affected by the presence of ascites or peripheral edema has also been recommended [22, 25]. Such parameters include mid-arm muscle circumference (MAMC), mid-arm circumference (MAC), and triceps skin fold thickness (TST). Diagnosis of malnutrition is established by values of MAMC and/or TST below the 5th percentile in patients aged 18–74 years, or the 10th percentile in patients aged over 74 years [26]. 

BMI changes may afford a reliable indicator of malnutrition using different BMI cutoff values depending on the presence and severity of ascites [26]; patients with a BMI below 22 with no ascites, below 23 with mild ascites, or below 25 with tense ascites are considered to be malnourished. Hand-grip examination by dynamometer has also been proposed as a simple method to detect patients at risk for the development of malnutrition [27]. In an interesting new development, Morgan et al. [28] validated a method where BMI and MAMC are combined with details of dietary intake in a semistructured algorithmic construct to provide a method for nutritional assessment in patients with end-stage liver failure [28]. Despite these advances, a standardized simple and accurate method for evaluating malnutrition in cirrhosis remains to be established.

## 5. Consequences of Cirrhosis with a Potential to Impact upon Nutritional Status and Brain Function

Cirrhosis results in multiple metabolic abnormalities and alterations in the synthesis, turnover, and elimination of a range of metal and micronutrients with the potential to alter nutritional status and consequently cerebral function. Such alterations include the following.

### 5.1. Hyperammonemia

**Figure 2:**
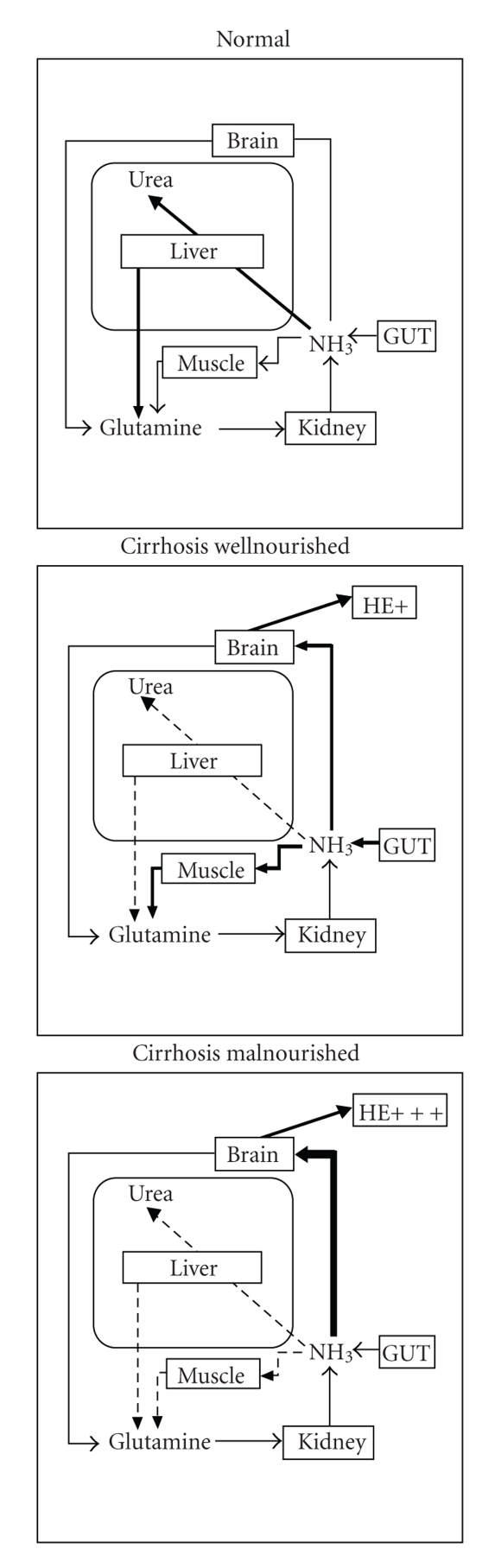
Inter-organ trafficking of ammonia in normal physiological conditions, in well-nourished patients with end-stage liver failure compared to malnourished end-stage liver failure patients.

Under normal physiological conditions, ammonia is metabolized by the liver, brain, muscle, and kidney (Figure 2). In well-nourished cirrhotic patients, the affected liver has an impaired capacity for removal of ammonia in the form of urea, which may result in increased muscle glutamine synthetase in order to provide an alternative mechanism for ammonia removal as glutamine. Glutamine synthesis also increases to some extent in the brain of these patients. HE may develop as a consequence of increased circulating and cerebral ammonia in well-nourished cirrhotic patients. On the other hand, in malnourished cirrhotic patients, the loss of muscle mass, commonly seen as a consequence of malnutrition, can adversely affect this alternative route of ammonia removal. The brain being the main organ metabolizing ammonia in these conditions, severe HE is commonly diagnosed in malnourished cirrhotic patients. 

Hyperammonemia may lead to increased uptake of tryptophan by the brain which may lead to increased synthesis and release of serotonin and anorexia. This symptom may render the patient prone to chronic catabolism and malnutrition, and in turn to increased ammonia load, resulting in a vicious cycle [29, 30]. In addition, hyperammonemia may be more prominent after gastrointestinal bleeding due to the absence of isoleucine [31]. Since haemoglobin molecule lacks the essential amino acid isoleucine, gastrointestinal bleed may stimulate the induction of net catabolism [32].

### 5.2. Zinc

Zinc is an essential trace element that plays an important role in the regulation of protein and nitrogen metabolism as well as in antioxidant defense. Reduced zinc content is common in cirrhotic patients, but zinc deficiency cannot be effectively diagnosed based upon serum concentrations since zinc is bound to albumin, which is also decreased in these patients [33, 34]. Among the mechanisms contributing to zinc deficiency, poor dietary intake [35], reduced intestinal absorption [36], reduced hepatointestinal extraction [37], portal-systemic shunting, and altered protein and amino acid metabolism have all been implicated [38]. Zinc deficiency may impair the activity of enzymes of the urea cycle as well as glutamine synthetase [39, 40], and decreased activity of these enzymes has the potential to lead to further increases in circulating and brain ammonia with the potential to cause worsening of HE. Not surprisingly, therefore, an inverse relationship between serum zinc and ammonia concentrations has been described [41, 42]. Zinc deficiency has been implicated in multiple complications of cirrhosis, including poor appetite, immune dysfunction, altered taste and smell, anorexia as well as altered protein metabolism [43, 44]. Surprisingly, in spite of evidence of hypozincemia in cirrhosis, zinc supplementation in the treatment of HE based on a small number of controlled trials has so far provided inconsistent results, a finding that may be attributable to variations in the nature and doses of zinc salts used and to duration of therapy [45].

### 5.3. Selenium

Decreased levels of selenium have been reported in cirrhotic patients [46, 47]. However, the relationship of diminished selenium to the pathogenesis of cirrhosis and its complications, including HE, has not been clearly established.

### 5.4. Manganese

In cirrhotic patients, the elimination of manganese is decreased secondary to impaired hepatobiliary function and portal-systemic shunting, which result in increased blood manganese levels and increased manganese deposition in basal ganglia structures of the brain, in particular in globus pallidus [48–52]. Manganese has also been correlated to increased brain glutamine levels [53] and changes in dopamine metabolism [49, 54] and may be related to other alterations in cirrhotic patients with HE, such as the characteristic astrocytic morphologic changes [55]. Toxic effects of manganese on central nervous system could be mediated by effects on the glycolytic enzyme glyceraldehyde-3-phosphate dehydrogenase (GAPDH) [56]. It was also suggested that manganese-induced increases of “peripheral-type” benzodiazepine receptors (PTBRs) could contribute to the pathogenesis of HE [57].

### 5.5. L-Carnitine

The liver is a major site for the production of ketone bodies from the oxidation of fatty acids. Fatty acids cannot penetrate the inner mitochondrial matrix and cross the mitochondrial membrane to undergo oxidation unless they are transported by a carrier process involving L-carnitine (3-hydroxy-4-trimethylammoniobutanoate). Carnitine is a cofactor for mitochondrial oxidation of fatty acids and prevents the body from using fats for energy production particularly during starvation. Carnitine deficiency may result in lethargy, somnolence, confusion, and encephalopathy. Studies of carnitine status in cirrhotic patients have yielded conflicting results; the source of this lack of consensus likely results from both the etiology of cirrhosis and the severity of liver disease. For example, Rudman et al. [58] reported reduced plasma and tissue carnitine concentrations in patients with alcoholic cirrhosis complicated by cachexia, whereas later studies by Fuller and Hoppel [59, 60] reported an increase of plasma carnitine in alcoholics with or without cirrhosis. De Sousa et al. [61] reported no such changes in a similar patient population. In a subsequent study by Amodio et al. [62], plasma carnitine levels were measured in cirrhotic patients and the relationship to nutritional status and severity of liver damage was assessed. Plasma carnitine levels did not differ between Child-Pugh class A, B, and C patients. Significantly higher levels of acetylcarnitine, short chain acylcarnitine, total esterified carnitine, and total carnitine were observed in cirrhotic patients independent of etiology of cirrhosis. The issue of carnitine in relation to liver disease was re-evaluated in 1997 by Krähenbühl and Reichen [63] who studied carnitine metabolism in 29 patients with chronic liver disease of varying degrees of severity and various etiologies. Patients with alcoholic cirrhosis manifested increased total plasma carnitine levels with a close correlation to serum bilirubin. Urinary carnitine excretion was not different between cirrhotic patients and controls with the exception of patients with primary biliary cirrhosis. It was concluded that patients with cirrhosis are not normally carnitine deficient and that patients with alcohol-induced cirrhosis manifest hypercarnitinemia which results primarily from increased carnitine synthesis due to increased skeletal muscle protein turnover [63].

### 5.6. Vitamin B_1_ (Thiamine)

Wernicke's Encephalopathy caused by vitamin B_1_ deficiency and characterized by a triad of neurological symptoms (ophthalmoplegia, ataxia, global confusional state) is common in cirrhotic patients. In a retrospective neuropathological study of sections from patients with end-stage liver failure who died in hepatic coma, 64% were found to manifest thalamic lesions typical of Wernicke's Encephalopathy [64]. None of the cases of Wernicke's Encephalopathy had been suspected based upon clinical symptoms during life, a finding which draws into question the classical textbook definition based upon symptomatology associated with the disorder [65].

Causes of vitamin B_1_ deficiency in cirrhosis include reduced dietary intake, impaired absorption, and loss of hepatic stores of the vitamin. Alcoholic cirrhotic patients manifest increased incidence of vitamin B_1_ deficiency compared to nonalcoholic cirrhotics [66]. Moreover, ethanol is known to impair both intestinal absorption of vitamin B_1_ [67] and to impair the transformation of the vitamin into its active (diphosphorylated) form [68]. It has been suggested that common pathophysiologic mechanisms exist in Wernicke's and hepatic encephalopathies, related to deficits of vitamin B_1_-dependent enzymes [69]. Vitamin B_1_ supplementation is highly recommended in patients with end-stage liver failure of either alcoholic or nonalcoholic etiologies.

## 6. Nutrition, HE, and Liver Transplantation

HE in end-stage liver failure may contribute to malnutrition in the pretransplant period as a consequence of diminished food uptake [70]. Alterations in markers of nutritional status such as serum albumin are significant risk factors for both surgical [71] and postsurgical [72] complications of liver transplantation. Moreover, it has been suggested that nonabsorbable disaccharides (such as lactulose) administered for the management of HE may result in intestinal malabsorption in patients with end-stage liver failure with the potential to result in poor transplant outcome [73]. 

The negative impact of malnutrition on liver transplantation had been reported in early retrospective studies [74]. Both preoperative hypermetabolism and body cell mass depletion proved to be of prognostic value for transplantation outcome [75]. Malnutrition is known to lead to glycogen depletion, and this has been suggested to increase the plasma lactate:pyruvate ratio during the anhepatic phase and to induce an exacerbated proinflammatory cytokine response, thereby favouring the development of postoperative systemic inflammatory response syndrome and multiorgan failure in these patients [76]. To date, there are still insufficient data in the pretransplant period upon which to base specific recommendations. In the posttransplant period, nutritional therapy improves nitrogen balance, decreases viral infection, and shows a trend to shortened intensive care unit stays with lowering of hospitalisation costs [77, 78].

## 7. Nutritional Recommendations for HE in End-Stage Liver Failure (Table 2)

### 7.1. General Considerations

Considering the high prevalence of malnutrition in cirrhotic patients together with the lack of simple and accurate methods of assessment of malnutrition in this patient population, it is reasonable to assume that malnutrition occurs in all patients. Nutritional requirements may vary according to the specific clinical situation. Multiple (5-6) small feedings with a carbohydrate-rich evening snack have been recommended with complex rather than simple carbohydrates used for calories. Lipids could provide 20%–40% of caloric needs. Long-term nutritional supplements may be necessary to provide recommended caloric and protein requirements. Additional studies are needed in order to formulate specific recommendations for nutrients such as zinc, selenium, and carnitine.

### 7.2. Energy Requirements

The primary goal for a patient suffering from end-stage liver failure should be to avoid by all means possible intentional or unintentional weight loss and sustain a diet rich in nutrients. It has been suggested that patients with liver cirrhosis should receive 35–40 kcal/kg per day [25].

### 7.3. Low Protein Diet to Be Avoided

Restriction of dietary protein was long considered a mainstay in the management of liver disease and HE [79, 80]. In particular, protein restriction (0–40 g protein/day) was shown to decrease encephalopathy grade in patients following surgical creation of a portal-systemic shunt, the only available therapy at one time for bleeding varices. Protein restriction (0–40 g protein/day) was later extended to include all patients with cirrhosis who developed encephalopathy. However, more recently, studies have shown that protein restriction in these patients has no impact on encephalopathy grade and that it may even worsen their nutritional status [81]. The increased awareness of the progressive deterioration of nutritional status in liver cirrhosis combined with a better understanding of metabolic alterations in the disorder has questioned the practice of prolonged protein restriction in the management of HE [82]. In fact, protein requirements are increased in cirrhotic patients, and high protein diets are generally well tolerated in the majority of patients. Moreover, the inclusion of adequate protein in the diets of malnourished patients with end-stage liver failure is often associated with a sustained improvement in their mental status. Furthermore, protein helps preserve lean body mass; this is crucial in patients with liver failure in whom skeletal muscle makes a significant contribution to ammonia removal. The consensus of opinion nowadays is that protein restriction be avoided in all but a small number of patients with severe protein intolerance and that protein be maintained between 1.2 and 1.5 g of proteins per kg of body weight per day. In severely protein intolerant patients, particularly in patients in grades III-IV HE, protein may be reduced for short periods of time [83–85].

### 7.4. Vegetable versus Animal Proteins

It has been suggested that vegetable proteins are better tolerated than animal proteins in patients with end-stage liver failure, a finding that has been attributed to either their higher content of branched-chain amino acids and/or because of their influence on intestinal transit [86, 87]. One study reported that a diet rich in vegetable protein (71 g/d) significantly improved the mental status of patients suffering from HE while increasing their nitrogen balance [88]. Vegetable proteins may also increase intraluminal pH and decrease gastric transit time. High dietary fibre diet has been recommended in order to abolish constipation which is an established precipitating factor for HE in patients with cirrhosis [89, 90]. A daily intake of 30–40 g vegetable protein has been found to be effective in the majority of patients [88].

### 7.5. Branched-Chain Amino Acids (BCAAs)

These amino acids (leucine, isoleucine, and valine) cannot be synthesized *de novo* but must be obtained from dietary sources and have a unique role in amino acid metabolism, regulating the intra- and interorgan exchange of nitrogen and amino acids by different tissues [91]. Chronic liver disease and portal-systemic shunting are characterized by a decrease in the plasma concentrations of BCAAs [92], whereas hyperammonemia increases their utilization. Since hyperammonemia results in increased utilization of BCAAs, which are largely metabolized by the muscle, it would be anticipated that providing BCAAs could facilitate ammonia detoxification by supporting muscle glutamine synthesis. Administration of BCAAs has been shown to stimulate hepatic protein synthesis; indeed, leucine stimulates the synthesis of hepatocyte growth factor by stellate cells [93]. Also, BCAAs reduce postinjury catabolism and improve nutritional status. Inadequate dietary protein intake or low levels of BCAAs may have a deleterious effect on HE [94], nutritional status [80], and clinical outcome [25, 81] in patients with end-stage liver failure. Clinical trials of BCAAs in the treatment of HE have yielded inconsistent findings. Several controlled clinical studies reported no efficacy of BCAAs on encephalopathy grade in patients with cirrhosis [95, 96]. However, other trials demonstrated that BCAAs were beneficial in similar patients [97, 98]. 

A double-blind, randomized clinical trial demonstrated that, in advanced cirrhosis, long-term nutritional supplementation with oral BCAA was useful to prevent progressive hepatic failure [99]. Furthermore, administration of solutions enriched with BCAAs has been shown to improve cerebral perfusion in cirrhotic patients [100]. Muto et al. [101] confirmed the beneficial effects of BCAAs using a more palatable granular formula. In a multicenter randomized study, it was also reported that long-term oral supplementation with a BCAA mixture improved the serum albumin level as well as cellular energy metabolism in cirrhotic patients [102]. 

The timing of BCAA supplementation in patients with end-stage liver failure may be crucial. This issue was addressed by a crossover study of 12 cirrhotic patients [103]. Daytime administration improved nitrogen balance and Fischer's ratio (ratio of BCAA/AAAs); however, both were further improved with nocturnal administration. At 3 months, a significant increase in serum albumin level was observed in patients administered nocturnal BCAAs, but not daytime BCAAs. It is possible that daytime BCAAs may be used primarily as calories, whereas nocturnal BCAAs may be preferentially used for protein synthesis. Furthermore, the long-term use of BCAAs in liver cirrhosis leads to an increase of serum protein of approximately 10% if given before bedtime [104]. Problems that limit the widespread use of BCAAs in the treatment of HE include their expense and unpalatability [105], both of which may result in poor patient compliance.

## 8. Antioxidants

### 8.1. Rationale for Use of Antioxidants

Cirrhotic patients manifest evidence of increased expression of biomarkers of oxidative stress such as increased lipid peroxidation [106, 107], as well as impaired antioxidant defences. Decreased levels of antioxidant micronutrients, including zinc [33, 107], selenium [46, 47], and vitamin E [107, 108] have been described in patients with end-stage liver failure. The potential benefits of vitamin E have been investigated, but results are conflicting. One randomized, placebo-controlled trial of vitamin E supplementation revealed a significant amelioration in terms of liver inflammation and fibrosis in patients with nonalcoholic steatohepatitis [109], while other studies with biochemical end points did not demonstrate any significant beneficial effect of vitamin E supplements [110]. In an earlier placebo-controlled randomized trial, 1-year vitamin E supplementation to patients with end-stage liver failure led to increased serum alpha-tocopherol levels, but did not result in any improvement in survival or quality of life [111]. The benefits of vitamin E therapy in relation to HE have not been assessed.

### 8.2. N-Acetylcysteine

A widely used complementary medical therapy for acute liver failure is the glutathione prodrug, N-acetylcysteine (NAC) [112, 113]. Glutathione is a major component of the pathways by which cells are protected from oxidative stress. NAC is an antioxidant with a thiol-containing compound and is used to restore cytosolic glutathione and detoxify reactive oxygen species and free radicals. NAC has proven beneficial in patients with type I hepatorenal syndrome [112] but was inefficient in patients with hepatitis C [113]. While NAC is widely used to treat acetaminophen hepatotoxicity, its benefit in end-stage liver failure with specific reference to HE remains to be established. In this regard, NAC is known to cross the blood-brain barrier and to improve central antioxidant status in the brain in mice with acute liver failure due to azoxymethane-induced hepatotoxicity [114].

## 9. Water-Soluble and Fat-Soluble Vitamins

Deficiencies in water-soluble vitamins (particularly the vitamin B complex) are common in end-stage liver failure [115]. A wide range of neuropsychiatric symptomatology associated with liver disease may be the consequence of water-soluble vitamin deficiencies. For example, peripheral neuropathy may result from pyridoxine, thiamine, or vitamin B_12_ deficiency. Confusion, ataxia and ocular disturbances are cardinal features of a lack of thiamine, and thiamine deficiency has been reported in patients with hepatitis C-related cirrhosis [116]. Deficiencies in vitamin B_12_, thiamine, and folic acid may develop faster in cirrhotic patients due to diminished hepatic storage. 

Fat-soluble vitamins (A, D, and K) deficiencies are likely to arise from malabsorption associated with end-stage liver failure. Vitamin A supplementation may be considered since vitamin A deficiency results in nyctalopia and dry cornea, and is associated with increased risk of hepatocellular carcinoma in patients with end-stage liver disease [117, 118]. Prescription of vitamin D, especially in patients with cholestasis (in combination with calcium since osteoporosis may be a complication of end-stage liver failure), is advised [118, 119]. Also, supplementation of vitamin K in conditions with high risk of bleeding such as the presence of impaired prothrombin time and oesophageal varices, should be considered [118]. In view of these findings, administration of multivitamin preparations is recommended.

## 10. Probiotics, Prebiotics, and Synbiotics

Probiotics are live microbiological dietary supplements with beneficial effects on the host beyond their nutritional properties. Prebiotics stimulate the growth and activity of beneficial bacteria within the intestinal flora. Synbiotics are a combination of pro- and prebiotics. Their mechanisms of action include the deprivation of substrates for potentially pathogenic bacteria, together with the provision of fermentation end products for potentially beneficial bacteria. Probiotic or prebiotic treatments aim at increasing the intestinal content of lactic acid-type bacteria at the expense of other species with more pathogenic potential. 

The concept of treating HE with probiotics was already suggested several decades ago [120–122]. The therapeutic benefit of acidifying the gut lumen with synbiotics in cirrhotic patients with minimal HE was demonstrated by Liu et al. [123] who showed that synbiotic/probiotic supplementation ameliorates hepatic function as reflected by reduced bilirubin and albumin levels and prothrombin times [123]. Modulation of gut flora was also associated with a significant reduction in blood ammonia levels and a reversal of minimal HE in 50% of patients [123]; improved hepatic function and serum transaminase levels in patients with alcohol- and hepatitis C-related cirrhosis have also been reported [124]. Another group reported improvement in biochemical and neuropsychological tests in cirrhotic patients receiving probiotics [125, 126]. Furthermore, liver transplant recipients who received a synbiotic regimen developed significantly fewer bacterial infections [127]. In a subsequent clinical trial, the incidence of postoperative bacterial infection as well as the duration of antibiotic therapy was significantly reduced in liver transplant patients receiving prebiotics [128]. More recently, Bajaj et al. [129] demonstrated a significant rate of minimal HE reversal in cirrhotic patients after probiotic yogurt supplements. Probiotics may provide additional benefits over dietary supplementation in reducing episodes of infection. Given the efficacy of probiotics and their lack of side effects, they are increasingly being used in the management of HE. 

## 11. Conclusion

Malnutrition is common in patients with end-stage liver failure and HE and adversely affects prognosis. Inadequate dietary intake, altered synthesis and absorption of nutrients, increased protein losses, hypermetabolism, and inflammation are among the factors contributing to malnutrition in this patient population. Although there are now several available methods to assess malnutrition, a standardized simple and accurate method for evaluating malnutrition in end-stage liver failure remains a challenge. Consequences of end-stage liver failure with a potential to impact upon nutritional status and brain function are numerous and include hyperammonemia, reduced zinc and selenium, manganese accumulation as well as deficiencies of carnitine and water-soluble vitamins, particularly thiamine. The primary goal for a patient with end-stage liver failure is to avoid by all means possible weight loss and sustain a diet rich in nutrients. A caloric intake of 35–40 kcal/kg/day is recommended. Low protein diets should be avoided and protein intake maintained at 1.2–1.5 g/kg/day. Particular attention should also be drawn to vegetable protein as well as to BCAAs which have proven beneficial in the treatment of HE. Antioxidants as well as probiotics are increasingly being employed in order to optimize the nutritional status in cirrhotic patients. Administration of multivitamin preparations, particularly thiamine, is recommended for patients with end-stage liver failure. Nutritional support to meet energy and substrate needs and to optimize the removal of circulating ammonia, reduce proinflammatory mechanisms, and improve antioxidant defenses has the potential to limit the progression of liver dysfunction, treat HE, and improve quality of life in patients with end-stage liver failure.

## Figures and Tables

**Table 1:** Metabolic alterations leading to malnutrition in end-stage liver failure.

Protein	Carbohydrate	Fat
(i) Increased catabolism (ii) Increased utilization of BCAAs (iii) Decreased ureagenesis	(i) Decreased hepatic and skeletal muscle glycogen synthesis (ii) Increased gluconeogenesis (iii) Glucose intolerance and insulin resistance	(i) Increased lipolysis (ii) Enhanced turnover and oxidation of fatty acids (iii) Increased Ketogenesis

**Table 2:** Nutritional recommendations for the management of HE in end-stage liver failure.

Substrate	Recommendation
Energy	35–40 kcal/kg/day
Protein	1.2–1.5 g/kg of body weight/day*
BCAA	In severely protein-intolerant patients
Antioxidant and vitamins	Multivitamin supplements
Probiotics, prebiotics	Increasing use for ammonia-lowering and anti-inflammatory actions

*In severely protein intolerant patients, protein may be reduced for short periods of time, particularly in grade III-IV hepatic encephalopathy.

## List of Abbreviations

AAA: Aromatic amino acids
BCAA: Branched-chain amino acid
BMI: Body mass index
GAPDH: Glyceraldehyde-3-phosphate dehydrogenase
HE: Hepatic encephalopathy
MAC: Mid-arm circumference
MAMC: Mid-arm muscle circumference
MELD: Model for end-stage liver disease
NAC: N-acetylcysteine
PTBR: Peripheral-type benzodiazepine receptor
SGA: Subjective global assessment
TNF-*α*: Tumor necrosis factor-alpha
TST: Triceps skin fold thickness.
